# Graft Survivals after Reconstruction Using Tumor-Bearing Frozen Bone in the Extremities

**DOI:** 10.3390/cancers15153926

**Published:** 2023-08-02

**Authors:** Shinji Miwa, Norio Yamamoto, Katsuhiro Hayashi, Akihiko Takeuchi, Kentaro Igarashi, Martin Louie S. Bangcoy, Yuta Taniguchi, Sei Morinaga, Yohei Asano, Hiroyuki Tsuchiya

**Affiliations:** Department of Orthopedic Surgery, Graduate School of Medical Sciences, Kanazawa University, Kanazawa 920-8640, Japan

**Keywords:** graft survival, reconstruction, frozen autograft, bone tumor, predictor

## Abstract

**Simple Summary:**

In this retrospective cohort study, predictive factors for graft survival were investigated in 123 patients who underwent reconstructions using a tumor-bearing frozen autograft after bone tumor resection of the extremities. The graft survival rates were 83.2% at 5 years and 70.2% at 10 years. Multivariate analysis using the Cox proportional hazards regression model revealed that BMI of ≥23.6 (HR, 3.4; *p* = 0.005), tibia (HR, 2.3; *p* = 0.047), and freezing procedure (HR, 0.3; *p* = 0.016) were independently associated with graft survival. Based on the results, pedicle or hemicortical freezing techniques are recommended in cases where these techniques can be applied.

**Abstract:**

Tumor-bearing frozen autografts have been widely used for reconstruction of bone defects caused by tumor resection. However, some patients undergo removal of the grafted bone due to surgical site infection, tumor recurrence, or fractures of the grafted bone. In this retrospective cohort study, predictive factors for graft survival were investigated in 123 patients who underwent reconstructions using a tumor-bearing frozen autograft after bone tumor resection of the extremities. To determine the independent predictors of graft survival, the association between various parameters and graft survival was investigated. The graft survival rates were 83.2% at 5 years and 70.2% at 10 years. Among the 123 frozen autografts, 25 (20.3%) were removed because of complications. In univariate analyses, male sex, BMI of ≥23.6, tibia, and chemotherapy were significantly associated with poor graft survival, whereas the pedicle/hemicortical freezing procedure was significantly associated with better graft survival. Multivariate analysis using the Cox proportional hazards regression model revealed that BMI of ≥23.6 (HR, 3.4; *p* = 0.005), tibia (HR, 2.3; *p* = 0.047), and freezing procedure (HR, 0.3; *p* = 0.016) were independently associated with graft survival. Based on the results, pedicle or hemicortical freezing techniques are recommended in cases where these techniques can be applied.

## 1. Introduction

The treatment of bone tumors involves tumor resection with appropriate surgical margins, chemotherapy, and radiation therapy. Reconstruction methods for bone defects after bone tumor resection are classified into endoprosthesis and biological reconstruction (allograft, tumor-bearing autograft treated with freezing, autoclaving, pasteurization, or irradiation) [1,2,3,4,5,6,7,8]. In biological reconstruction, good functional outcomes can be expected owing to good attachment between the grafted bone and soft tissues, such as muscles and tendons. Currently, tumor-bearing autografts treated with irradiation, pasteurization, or liquid nitrogen are widely used in the reconstruction of bone defects after bone tumor resection [2,3,5,9,10,11]. Frozen autografts have several advantages, including good osteoinduction, osteoconduction, simple procedure, short operative time, and revitalization [9,12,13,14]. Furthermore, various techniques, such as pedicle freezing, hemicortical freezing, and composite use of prostheses, have been developed to prevent freezing-related postoperative complications, such as fracture, delayed union, and osteoarthritis [7,10]. On the other hand, some patients require the removal of the frozen bone due to postoperative complications such as surgical site infection (SSI), tumor recurrence, or fractures of the grafted bone. To identify appropriate procedures and indications for tumor-bearing frozen autografts, information about predictive factors for graft survival is important. In this study, graft survival and its predictive factors were investigated in patients who underwent reconstruction with a tumor-bearing frozen autograft after bone tumor resection in the extremities.

## 2. Materials and Methods

### 2.1. Patients

Between January 1999 and August 2022, 139 patients with bone tumors in the extremities underwent tumor resection and reconstruction using a tumor-bearing frozen autograft. All patients or guardians provided written informed consent at the time of admission for inclusion in the scientific studies. This retrospective cohort study included 123 patients based on the following inclusion criteria: (1) histologically confirmed bone tumors, (2) located in the extremities, (3) reconstruction using a frozen autograft, and (4) follow-up period of ≥6 months (Table 1). Five patients were excluded from the study because of incomplete information regarding the clinical parameter or type of treatment. Eleven patients with a follow-up period of <6 months were excluded from this study. There were 61 males and 62 females (median age 25 years, range 6–90). The tumor location was the femur in 71 patients, the tibia in 35 patients, the humerus in 16 patients, and the radius in 1 patient. There were 94 primary and 29 metastatic bone tumors. Primary bone tumors comprised the following: osteosarcoma in 70 patients, chondrosarcoma in 7 patients, Ewing’s sarcoma in 5 patients, undifferentiated pleomorphic sarcoma/malignant fibrous histiocytoma in 5 patients, leiomyosarcoma in 2 patients, adamantinoma in 1 patient, myeloma in 1 patient, fibrosarcoma in 1 patient, aggressive osteoblastoma in 1 patient, and fibroblast growth factor 23 producing tumor in 1 patient. All tumor specimens were histologically diagnosed by pathologists at our hospital. 

The associations among various parameters, including age, sex, body mass index (BMI), tumor location (femur, tibia, or upper extremity), tumor histology (primary or metastasis), chemotherapy, freezing method (free freezing, pedicle freezing, or hemicortical freezing), type of implant (prosthesis, plate, intramedullary nail, screw, and combination of these implants), length of the frozen bone, operative time, intraoperative blood loss, and graft survival were investigated. Graft survival was defined as the time from the day of the operation to the day of removal of the tumor-bearing frozen autograft. 

This retrospective study was approved by the Medical Ethics Committee of our institute (Institutional Review Board [IRB] number, 2022-202). Informed consent was obtained using the opt-out method, and the written informed consent was waived by the IRB. This study was registered in the Japan Registry of Clinical Trials. The work has been reported in line with the STROCSS criteria [15].

### 2.2. Surgical Procedure

**Figure 1:**
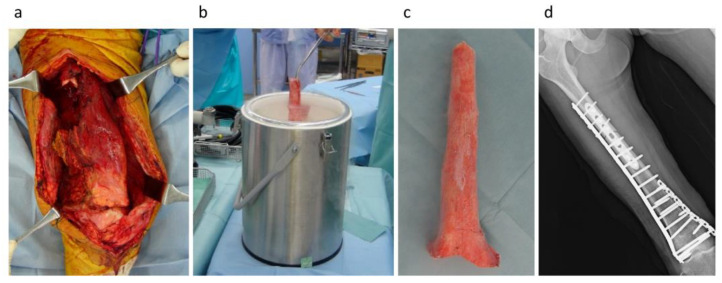
Free-freezing procedure. (**a**) The tumor was excised with appropriate surgical margin. (**b**) The tumor-bearing bone was frozen in liquid nitrogen after curettage and drilling. (**c**) Tumor-bearing frozen bone. (**d**) After the freezing, tumor-bearing frozen bone was used for reconstruction.

**Figure 2:**
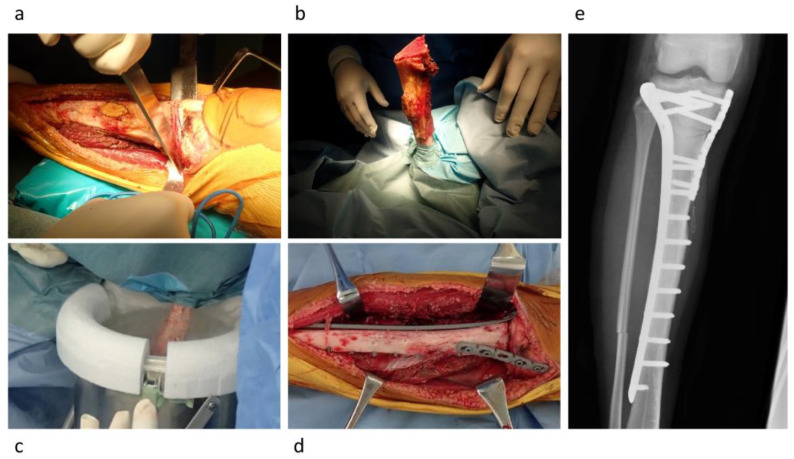
Pedicle freezing procedure. (**a**) Osteotomy was performed in the proximal site with surgical margin. (**b**) The tumor-bearing bone was isolated by surgical sheets. (**c**) The tumor-bearing bone was frozen in liquid nitrogen by rotation of the affected limb. (**d**) After the freezing, the tumor-bearing frozen bone was used for reconstruction. (**e**) Postoperative X-ray.

**Figure 3:**
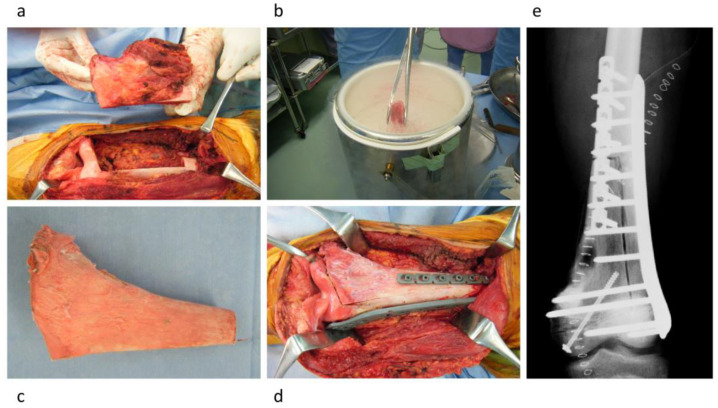
Hemicortical freezing procedure. (**a**) Hemicortical resection of the tumor. (**b**) Freezing in liquid nitrogen. (**c**) Tumor-bearing frozen bone. (**d**) The tumor-bearing frozen bone was used for reconstruction of the bone defect. (**e**) Postoperative X-ray.

The freezing procedure was performed using the following steps: freezing the tumor-bearing bone in liquid nitrogen for 20 min, thawing at room temperature for 15 min, and thawing in distilled water for 10 min [9]. Freezing procedures were classified into (1) free freezing, (2) pedicle freezing, and (3) hemicortical freezing [7,9,10,16]. The free-freezing procedure included excision of the bone lesion by bicortical osteotomy with an appropriate surgical margin, curettage of the bone lesion, and freezing in liquid nitrogen (Figure 1). The pedicle freezing procedure involved exposure of the bone lesion using either proximal or distal osteotomy, prevention of tumor contamination by utilizing surgical sheets, curettage of the bone lesion, and freezing in liquid nitrogen (Figure 2). The hemicortical freezing procedure comprised hemicortical resection of the tumor in cases with eccentric tumor location in the long bone, curettage of the lesion, and freezing in liquid nitrogen (Figure 3). Tumor-bearing frozen bone was reconstructed using plates, intramedullary nails, or composites using prostheses. Curetted tumor tissues were histologically evaluated after surgery. Surgical drains were removed when the amount of drainage was ≤50 mL, and intravenous antibiotic administration was continued until the drain tube was removed. All of the surgical procedures were performed by surgeons with more than 15 years of experience.

### 2.3. Statistical Analyses

Statistical significance was set at *p* < 0.05. The optimal cutoff levels of age, BMI, length of the frozen bone, operative time, and intraoperative blood loss were determined using time-dependent receiver operator characteristic analysis. Graft survival was calculated and compared using the Kaplan–Meier method with the log-rank test. To identify independent predictors of graft survival, multivariate analysis using the Cox proportional hazards regression model was performed with graft survival time as the dependent variable. All parameters with *p* < 0.05 in the univariate analyses were included in the Cox proportional hazards regression model. EZR statistical software (Saitama Medical Center, Jichi Medical University, Saitama, Japan) was used to perform all the statistical analyses.

## 3. Results

The mean length of the grafted bone was 13.7 ± 6.1 cm. The 5- and 10-year graft survival rates were 83.2% and 70.2%, respectively. Among the 123 frozen autografts, 25 (20.3%) were removed because of complications, including infections in 12 (9.8%), local recurrences in 8 (6.5%), and fractures in 5 (4.1%). In patients who underwent graft removal, the mean time to graft removal was 49.3 (range, 1–150) months. 

**Figure 4:**
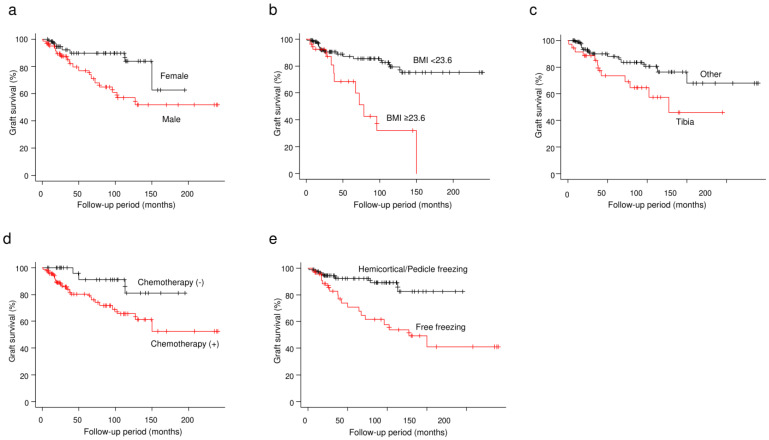
The Kaplan−Meier curves show the graft survivals. (**a**) Gender. (**b**) BMI. (**c**) Tumor location. (**d**) Chemotherapy. (**e**) Freezing procedure.

In the univariate analyses, male sex, BMI of ≥23.6, tibial tumor, and chemotherapy were significantly associated with poor graft survival, whereas pedicle/hemicortical freezing procedures were associated with better graft survival (Table 2; Figure 4). The 5-year graft survival rates in males and females were 76.8% and 89.6%, respectively (*p* = 0.017). The 5-year graft survival rates in patients with BMIs of ≥23.6 and <23.6 were 68.4% and 87.1%, respectively (*p* < 0.001). The 5-year graft survival rates were 100% in the upper extremities, 84.4% in the femur, and 73.4% in the tibia. Graft survival in patients with tibial tumors was significantly lower than in those with tumors located at other sites (*p* = 0.034). The 5-year graft survival rates in patients who underwent chemotherapy and those who did not were 80.3% and 91.1% (*p* = 0.033), respectively. The 5-year graft survival rates were 100% in patients who underwent hemicortical freezing, 93.6% in those who underwent pedicle freezing, and 70.6% in those who underwent free freezing. The hemicortical/pedicle freezing procedure had significantly better graft survival than the free-freezing procedures (*p* = 0.001). The 5-year graft survival rates were 88.8% in reconstruction using plates, 85.1% in frozen autograft-prosthesis composite reconstruction, 71.1% in reconstruction using intramedullary nails, and 75.0% in other or combination use of implants. Although the use of intramedullary nails resulted in a lower graft survival rate, no significant difference was observed (*p* = 0.077). Age, tumor histology, length of the frozen autograft, operative time, and intraoperative blood loss were also not significantly associated with graft survival.

To identify independent predictors for graft survival after reconstruction using tumor-bearing frozen autografts, male sex, BMI of ≥23.6, tibia, chemotherapy, and freezing procedure were included in the Cox proportional hazards regression models (Table 3). Multivariate analysis revealed that a BMI of ≥23.6 (HR, 3.4; *p* = 0.005), tibia (HR, 2.3; *p* = 0.047), and hemicortical/pedicle freezing procedure (HR, 0.3; *p* = 0.016) were independent predictors of graft survivals.

## 4. Discussion

In studies on reconstructive surgery, various biological materials including skin, acellular dermal matrix (biological collagen matrix), allograft, iliac or fibular graft, and devitalized bone graft have been reported [17,18]. Although allograft is one of the useful biological materials, there is a vital deficit of donors and the reserves of allografts are not sufficient. The key role in the success of the allogenic transplantation is to the broad and systematic education of the society [19]. Among the biological materials, tumor-bearing frozen autograft has been used for reconstruction of bone tumors. In this study, the 5- and 10-year graft survival rates after reconstruction with frozen autografts were 83% and 70%, respectively. In previous studies, the long-term graft survival rates after biological reconstruction have been reported to be 56–86% [8,20,21,22,23]. Aponte-Tinao, et al. investigated graft survival after reconstruction using allograft [8]. In their study, the graft survival rates were 74% at 5 years, 60% at 10 years, and 56% at 20 years. Crenn et al. reported that the 5-year graft survival rate was 71% after reconstruction using an allograft without a vascularized fibula graft [24]. In another study of allograft reconstruction for the humerus, the 5-year graft survival rate was 71% [25]. Puri et al. reported a 5-year graft survival rate of 79% in 70 patients treated with intercalary reconstruction using irradiated bone [22]. Lee et al. investigated clinical outcomes in 278 patients who underwent reconstruction using pasteurized bone and reported that the graft survival rates were 73% at 5 years, 59% at 10 years, and 40% at 20 years [26]. In another study of 14 patients with pasteurized bone grafts, the 5- and 10-year graft survival rates were 79% and 48%, respectively [27]. Song et al. reported a 10-year graft/implant survival rate of 69% in 25 patients who underwent pasteurized autograft-prosthesis composite reconstruction [28]. In the present study, the graft survival rates were 100% in hemicortical freezing and 94% in pedicle freezing, which were higher than those in the other devitalized autografts. The reasons for the higher graft survival rates after hemicortical reconstruction are thought to be the wide contact area and mechanical support by cancellous and cortical bone on the other side of the involved long bone [7]. In contrast, the pedicle freezing procedure has several advantages, including a decreased osteotomy site and shorter operative time than the free freezing procedure, which may contribute to low rates of complications such as non-union, mechanical failure, and infection [10]. In a retrospective study comparing pedicle freezing procedure and free freezing procedure, the union period was shorter and the rate of postoperative complications was lower with the PFP than with the FFP [29]. Based on these studies, hemicortical reconstruction or pedicle freezing techniques are recommended in cases in which these procedures can be applied. The incidences of non-union were reported to be 14–47% after intercalary reconstruction, 6% after reconstruction with pedicle freezing procedure, and 0% after hemicortical reconstruction [7,10,30,31]. Frisoni et al. investigated the risk factors for non-union after reconstruction using allograft [32]. In their study, the use of intramedullary nails, adjuvant chemotherapy, length of resection (≥17 cm), and age (≥18 years) negatively affected the outcomes. In our study, chemotherapy and the use of intramedullary nails were associated with poor graft survival in the univariate analysis, although the univariate and multivariate analyses did not show a significant association between these factors and graft survivals. Further studies are required to evaluate the association between these factors and graft survival.

Previous studies have reported an association between BMI and postoperative infection. In a meta-analysis study on the risk factors for postoperative infection after spine surgery, BMI and diabetes were associated with an increased risk of postoperative infection [33]. In another study involving 32 patients who underwent proximal tibial tumor resection and reconstruction using allograft, BMI was significantly associated with postoperative deep infection [34]. In obese patients who undergo reconstruction using frozen autografts, the use of antibacterial implants or extended use of antibiotics is recommended. Another possible reason for poor graft survival in patients with a higher BMI is thought to be due to weight loading on the grafted bone. Weight loading may cause instability, delayed union, fracture, and collapse of the grafted bone, although no study has shown a significant association between BMI and the structural failure in patients who underwent biological reconstruction. 

In other studies, high failure rates were reported in patients who underwent reconstruction in the tibia [35,36]. In a study on risk factors for SSI after malignant tumor resection and reconstruction in the extremities, tibial tumor and long operative time were independently associated with an increased risk of SSI [37]. Compared with other sites, soft tissue covering may have been insufficient after reconstruction after tibial tumor resection, and the limited soft tissue covering may have caused infection or delayed union due to insufficient blood flow. Reconstruction after tibial tumor resection requires appropriate soft tissue covering, and flap procedures should be discussed in cases of insufficient soft tissue covering.

This study has several limitations, including the small number of study patients, heterogeneous sites, histology, implants, and reasons for graft removal. Because this study focused on patients who underwent reconstruction using frozen autografts, only 123 patients were included in the analyses. In this study, the reasons for removal of frozen autografts included infections, local recurrences, or fractures. Risk factors for each complication should be investigated in future studies. Furthermore, most of the study patients were Japanese, and the BMI and bone size of these patients may differ from those of patients of other races. To elucidate the predictors of graft survival in other races, further studies in various races are required.

## 5. Conclusions

In summary, the present study showed a BMI of ≥23.6 (HR, 3.4; *p* = 0.005), tibia (HR, 2.3; *p* = 0.047), and freezing procedure (HR, 0.3; *p* = 0.016) were independent predictors of graft survival. Pedicle freezing and hemicortical freezing are recommended in cases where these techniques can be applied. 

## Figures and Tables

**Table 1:** Patient characteristics.

Variable	All Patients (n = 123)
Sex	
Male	61 (50%)
Female	62 (50%)
Mean age at diagnosis (years)	35.2 ± 23.4
Mean follow-up period (months)	80.8 ± 64.4
Histologic diagnosis	
Primary tumors	94 (76%)
Metastatic tumors	29 (24%)
Location	
Femur	71 (58%)
Tibia	35 (28%)
Humerus	16 (13%)
Radius	1 (1%)
Chemotherapy	98 (80%)
Freezing technique	
Free freezing	45 (37%)
Pedicle freezing	71 (58%)
Hemicortical freezing	7 (6%)
Implant	
Prosthesis	46 (37%)
Plate	45 (37%)
Intramedullary nail	26 (21%)
Prosthesis and plate	2 (1%)
Prosthesis and nail	1 (1%)
Plate and nail	1 (1%)
External fixation	1 (1%)
Screw	1 (1%)
Length of the frozen autograft (cm)	13.7 ± 6.1
Intraoperative blood loss (mL)	349.0 ± 294.4
Operative time (min)	348.1 ± 105.2
Graft survival	98 (80%)

**Table 2:** Univariate analysis of factors affecting graft survival.

Variable	No. of Patients (n = 123)	5-Year Graft Survival (%)	95% CI	*p* Value
Sex				0.017
Male	61 (50%)	76.8	61.5–86.7	
Female	62 (50%)	89.6	76.6–95.6	
Age (years)				0.870
≥38	33 (27%)	82.1	63.0–92.0	
<38	90 (73%)	83.7	71.6–90.9	
BMI				<0.001
≥23.6	27 (22%)	68.4	41.6–84.8	
<23.6	96 (78%)	87.1	77.1–92.9	
Site				0.034
Tibia	35 (28%)	73.4	53.3–85.9	
Others	88 (72%)	87.8	76.9–93.8	
Histology				0.223
Primary tumor	95	80.6	69.7–87.9	
Metastatic tumor	28	96.4	77.2–99.5	
Chemotherapy				0.033
Yes	90 (73%)	80.3	68.6–87.9	
No	33 (27%)	91.1	68.8–97.7	
Freezing technique				0.001
Free freezing	45 (37%)	70.7	53.0–82.7	
Hemicortical/pedicle	78 (63%)	92.1	81.8–96.7	
Implant				0.077
Intramedullary nail	26 (21%)	71.1	45.8–86.2	
Others	97 (79%)	86.5	76.0–92.6	
Length of the frozen autograft				0.119
≥115 mm	68 (55%)	77.7	63.8–86.8	
<115 mm	55 (45%)	90.4	75.8–96.4	
Operative time				0.051
≥342 min	56 (46%)	71.2	54.1–82.9	
<342 min	67 (54%)	92.3	80.5–97.0	
Intraoperative blood loss				0.141
≥170 mL	87 (71%)	80.7	68.8–88.5	
<170 mL	36 (29%)	89.3	70.1–96.5	

**Table 3:** Independent predictors for graft survival in the Cox proportional hazards regression models.

Factor	Hazard Ratio	95% CI	*p* Value
Male	2.047	0.844–4.969	0.113
BMI ≥ 23.6	3.350	1.237–8.047	0.005
Tibia	2.290	1.011–5.187	0.047
Chemotherapy	1.886	0.522–6.811	0.333
Hemicortical/pedicle freezing	0.317	0.124–0.809	0.016

## Data Availability

The datasets supporting the conclusion of this article are included within the article. The underlying datasets are available from the corresponding author upon request.
